# Editorial

**Published:** 1983-07

**Authors:** 


### EDITORIAL

Number 1 of Volume 1 of our *Journal* is dated July 1883 and this number dated July 1983 is the true Centenary number. As in the January number beginning our Centenary year we have two weighty historical articles. The first on Ernest Hey Groves by Mr. A. H. Ratcliffe was given to the Society at the Autumn Clinical Meeting of the Society in 1981 at the Bristol Royal Infirmary and was a repeat of his Robert Jones Lecture delivered at the Royal College of Surgeons. It is a fascinating account of an extraordinary man and perhaps the greatest figure on the Bristol surgical scene. For the second, Dr. Patricia Craig has researched the first meeting of the British Medical Association (then the Provincial Medical and Surgical Association) in Bristol 150 years ago. If Medicine has travelled a long way since our first number when men were arguing about the cause of Phthisis, during that 150 years it has come from the Middle Ages, when Dr. Edward Barlow of Bath, one of the principal speakers could state that the recent cholera epidemic was an Act of God which doctors should not have been expected to control – 'to arrest the course of such dispensation is as hopeless as it is irrational' – and yet how tempting now it is to see the appearance of a new and fatal disease, Acquired immune deficiency syndrome (AIDS) amongst colonies of Male Homosexuals in the USA as a modern version of the destruction of Sodom and Gomorrah.

**Membership of the Society**

The first number contained a list of subscribers to the *Journal* of whom there were 292. In 1933 the list of subscribers was also printed and this time there were 284, surprisingly a reduction. We print in this number a list of current members of the Society (446 with 43 independent subscribers) not only to keep a tradition going but also to enable members to identify non-members and persuade them to join. Our present membership brings the *Journal* an income of about £1000 per quarter. It costs £1092 to print a number with 48 pages. Advertisements are hard to come by and constantly being sought. For the continued health of the *Journal* more members are most desirable and these have to be proposed by existing members. If members will look through the list and consider whether they have colleagues who are not on our list and might like to become members, and if they can persuade them to join, either as full members (£12 p.a.) or subscribers to the *Journal* (£8 p.a.) they will be doing the Society a service.

**Gardner's World**

It has always been the aim of this *Journal* to attract contributions from other centres in the South West of England. In this number we have three articles from Mr. Michael Gardner of Torquay and currently President of the South West Surgeons Club, two of these are on basic physiology and one, on the human foot pump is an original observation. He has shown that not only do the calf muscles act as a pump returning blood from the leg, but that a pump also exists in the sole of the foot, operated by the patient's weight. Mr. Gardner has been active over many years with his colleagues in Torquay in pursuing research both clinical and fundamental. His many writings record his work at Torquay with no resources other than ingenuity and enthusiasm. We applaud his work and are privileged in this instance to print an original report of a fundamental body mechanism previously unrecognised.

